# Adenoviral vectors for highly selective gene expression in central serotonergic neurons reveal quantal characteristics of serotonin release in the rat brain

**DOI:** 10.1186/1472-6750-9-23

**Published:** 2009-03-19

**Authors:** Kheira Benzekhroufa, Beihui Liu, Feige Tang, Anja G Teschemacher, Sergey Kasparov

**Affiliations:** 1 Department of Physiology and Pharmacology University of Bristol, BS8 1TD, UK

## Abstract

**Background:**

5-hydroxytryptamine (5 HT, serotonin) is one of the key neuromodulators in mammalian brain, but many fundamental properties of serotonergic neurones and 5 HT release remain unknown. The objective of this study was to generate an adenoviral vector system for selective targeting of serotonergic neurones and apply it to study quantal characteristics of 5 HT release in the rat brain.

**Results:**

We have generated adenoviral vectors which incorporate a 3.6 kb fragment of the rat tryptophan hydroxylase-2 (TPH-2) gene which selectively (97% co-localisation with TPH-2) target raphe serotonergic neurones. In order to enhance the level of expression a two-step transcriptional amplification strategy was employed. This allowed direct visualization of serotonergic neurones by EGFP fluorescence. Using these vectors we have performed initial characterization of EGFP-expressing serotonergic neurones in rat organotypic brain slice cultures. Fluorescent serotonergic neurones were identified and studied using patch clamp and confocal Ca^2+ ^imaging and had features consistent with those previously reported using post-hoc identification approaches. Fine processes of serotonergic neurones could also be visualized in un-fixed tissue and morphometric analysis suggested two putative types of axonal varicosities. We used micro-amperometry to analyse the quantal characteristics of 5 HT release and found that central 5 HT exocytosis occurs predominantly in quanta of ~28000 molecules from varicosities and ~34000 molecules from cell bodies. In addition, in somata, we observed a minority of large release events discharging on average ~800000 molecules.

**Conclusion:**

For the first time quantal release of 5 HT from somato-dendritic compartments and axonal varicosities in mammalian brain has been demonstrated directly and characterised. Release from somato-dendritic and axonal compartments might have different physiological functions. Novel vectors generated in this study open a host of new experimental opportunities and will greatly facilitate further studies of the central serotonergic system.

## Background

Animal and human studies have associated the serotonergic (5 HTergic) system with diseases such as depression and anxiety [1-3], or schizophrenia [4,5]. Drugs which modulate 5 HT re-uptake are widely used for treatment of depression and the range of their clinical applications is expanding [6,7].

Surprisingly little is known about the neurobiology of central 5 HTergic neurones because these cells are intermingled with other cellular phenotypes even in nuclei where their density is highest. Hence, while numerous experimental and clinical studies point to 5 HT release and uptake as highly medically relevant processes [6,8], it has been impossible to directly study these processes at single cell level. In recent years, targeted expression of genes to selective cellular phenotypes has become a popular strategy for studies of brain function. We reasoned that a method for selective gene expression and manipulation in 5 HTergic neurones would be extremely useful for understanding the mechanisms by which 5 HT exerts its actions. Indeed, even an ability to directly visualize 5 HTergic neurones live within the highly heterogeneous cell milieu of the brain would offer a whole host of opportunities for electrophysiological, imaging, amperometry and other studies [9].

Two strategies may be used for selective targeting – germline transgenesis or viral vectors. Both approaches rely on the use of a cell-specific promoter (CSP) which is able to drive cell type-specific transgene expression. Transgenic mice have been developed where the enhancer region of the transcription factor Pet-1 was used to drive either Cre-recombinase (Cre) or yellow fluorescent protein expression in 5 HTergic neurones [10,11]. Using Pet-1 for expressing Cre in mice with floxed allele of another transcription factor, Lmx1b, it is possible to selectively suppress 5 HTergic neurones in mice [12,13]. These mice models are a valuable resource suitable for some types of experiments. For a number of years several groups have been pursuing a different strategy, namely using viral vectors for gene expression in selected cellular groups in the brain [14-18]. One great advantage of viral transgenesis is that it is independent of the animal strain, for instance the same vector has been used to visualise catecholaminergic neurones in normo- and hypertensive rats [19]. Moreover, as critical sequences in promoters seem to be highly conserved, the same CSPs usually work equally well cross-species. For example, the synapsin-1 (SYN) promoter which is commonly used for viral gene expression in rodent studies was originally cloned from human DNA [16], and the human and mouse promoter sequences of glial fibrillary acidic protein are active and cell-specific in both rats and mice [18,20]. Therefore, with viral vectors, specific gene manipulation becomes possible in various normal and mutant lines of rats, mice and probably many other species. This becomes particularly relevant in research areas where rat models are of critical value, for example in anxiety-and depression-related studies [21,22]. Viral vectors are also highly versatile, easily transferable between laboratories and relatively inexpensive to produce. Finally and importantly, because the viral vectors can be applied to the postnatal tissue, it is possible to achieve much higher levels of transgene expression than with any standard germline transgenic approach without the risk of embryonic toxicity.

We set out to develop a viral vector system for selective targeting of central 5 HTergic neurones. First, we explored the regions upstream of the coding sequence for tryptophan hydroxylase-2 (TPH-2), the key enzyme in central neuronal 5 HT synthesis [23] and found 2 kb – 3.6 kb of the promoter region to confer selective expression to raphe 5 HTergic neurones [24]. Because these sequences were insufficiently powerful to drive visible expression of a fluorescent marker such as enhanced green fluorescent protein (EGFP) we potentiated CSP using a two-step transcriptional amplification (TSTA) strategy [24-27]. This strategy is based on simultaneous cell-specific expression of artificial transcriptional enhancers and their unique binding domains which are placed upstream of a weak CSP [20,27,28].

The lentiviral vector (LVV) generated in our previous study [24] will be a valuable resource, predominantly for *in vivo *experimentation on the 5 HTergic system. However, for many types of *in vitro *experiments, adenoviral vectors (AVV) are preferable since they are much more efficient in slice cultures and cheap to generate in large quantities [15]. The objective of the current study was therefore two-fold. First, building on the success of our previous work, we set out to generate a TSTA-amplified AVV system for specific targeting 5 HTergic neurones in organotypic slice cultures *in vitro*. Second, we applied this system to obtain the first information about the characteristics of quantal 5 HT release from 5 HTergic neurones of mammalian brain.

## Results

The ability to visualize 5 HTergic neurones in living brain tissue preparations, rather than via post-hoc immunohistochemical identification or other less direct means of phenotype verification opens a wide range of opportunities for investigation of cellular physiology and signalling properties of these cells. Organotypic brain slice cultures transduced with AVV are excellent models for confocal imaging and electrophysiological experiments [15,29,30]. Previously, we have successfully used this approach for studies of GABAergic and catecholaminergic neurones in the brainstem [17,19,31].

**Figure 1:**
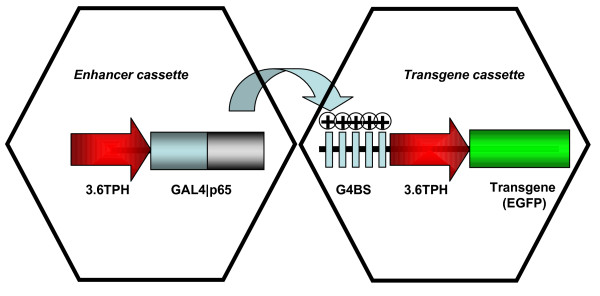
**Binary AVV system based on 3.6 TPH promoter and TSTA**. In this design, the first AVV utilises 3.6 TPH to express the chimeric recombinant transcriptional enhancer, GAL4|p65. The second AVV contains the actual transgene under control of the 3.6 TPH promoter but with G4BS (multimerised GAL4 binding sites) fused to its 5' end. Binding of the enhancer to these sites strongly potentiates the expression of EGFP.

**Figure 2:**
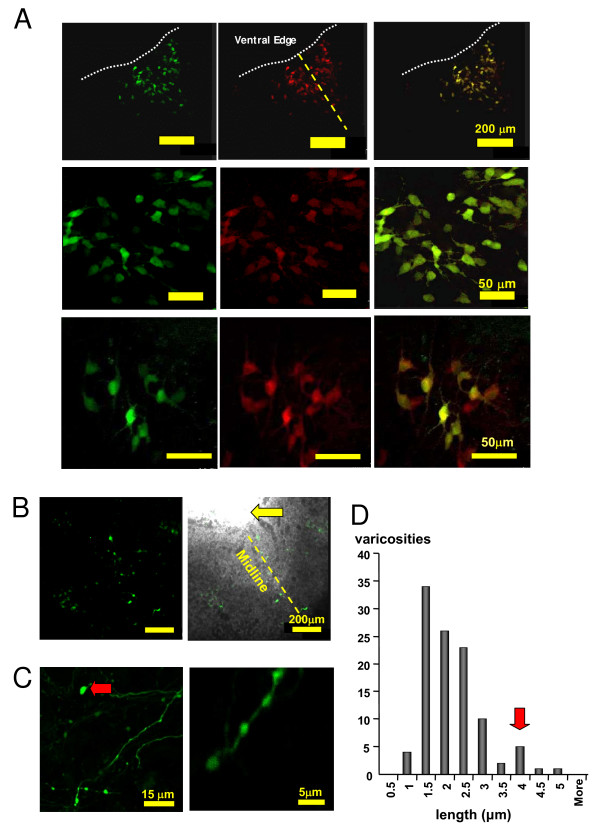
**Selective EGFP expression in raphe 5 HTergic neurons in organotypic slice cultures**. **A**. Confocal images from slice cultures of the dorsal raphe after transduction with the dual AVV system (AVV-G4BS-3.6TPH-EGFP and AVV-3.6TPH-GAL4p65) and immunostained for TPH-2 (centre panels). EGFP expression (left panels) was 5 HT-selective (overlay of green and red channel – right panels) and sufficiently potent for single-cell experiments on live 5 HTergic neurones. Middle row of images in a higher power image of the upper one. Lower row of images is from a different slice, closer to the ventral edge (white dotted line). Yellow dashed line in upper raw shows approximate midline. **B**. Distribution of EGFP-expressing 5 HTergic neurones in a midbrain slice culture. Left panel – EGFP fluorescence, right panel – overlay of fluorescence channel on the DIC image. Yellow arrow points at the edge of the aqueduct. Cells are largely located along midline (B7/B8 groups), some more lateral cells probably come from B9 group. **C**. Characteristic varicosities of EGFP-expressing 5 HTergic axons. Note that varicosities are predominantly small but few of a larger type can also be observed (red arrow). **D**. Morphometric analysis of living 5 HTergic varicosities (n = 106) in slice cultures. The histogram shows the distribution of varicosity lengths measured along the axis of the axon. While the majority of varicosities were small (1.5–2 μm) there was a distinct sub-population of larger varicosities (median ~4 μm, red arrow). Large varicosities are rare, but could represent a specific functional phenotype.

In our recent study, we used LVV *in vivo *to determine those regions upstream of the TPH-2 coding sequence which confer 5 HTergic neurone-specific expression [24]. TSTA-amplified 3.6 kb promoter region (3.6 TPH) resulted in visible EGFP fluorescence in living 5 HTergic neurones which was nearly 100% specific in the brainstem areas tested. Due to the size of 3.6 TPH in relation to AVV capacity (<8 kb), in this study, we decided to use the two-promoter-copy strategy incorporated into two separate AVV (Figure 1 see [24]. We previously used similar two-vector approaches, e.g. [20,28] and know that transduced cells take up multiple copies of the viral genomes, which ensures that both parts of the system are in place. In the present experiments we employed AVV-G4BS-3.6TPH-EGFP and AVV-3.6TPH-GAL4|p65 to transduce brainstem slice cultures. Similar to the corresponding LVV described in [24], the dual AVV system demonstrated high specificity. 97% of EGFP-expressing neurones in slice cultures were also TPH-2-positive (estimate based on n~700 cells from 20 slice cultures from 4 separate preparations; Figure 2A–B). Fluorescence was clearly detectable after 2 – 3 days and reached an apparent plateau after 6–7 days. Several dozens of fluorescent neurones could be visualised in each healthy slice. We did not evaluate the duration of expression in this study but from our previous experience with AVV carrying CSP it is likely to last for the duration of the life of the slice culture. No EGFP fluorescence was evident in cultures transduced with AVV 3.6TPH-EGFP alone. Not only cell somata, but also numerous axons with characteristic varicosities could be visualized (Figure 2C). High power confocal stacks of images of living EGFP-expressing varicosities were collected and analyzed post-hoc. The absolute majority of living 5 HTergic varicosities (92% of 106 measured) were elongated (average aspect ratio 1.46) along the axonal axis and very small (median ~1.5 μm along the long axis; Figure 2C–D). Interestingly, there was also a distinct minority population (~10%) of larger varicosities with medial length ~4 μm (Figure 2C–D, red arrows).

**Figure 3:**
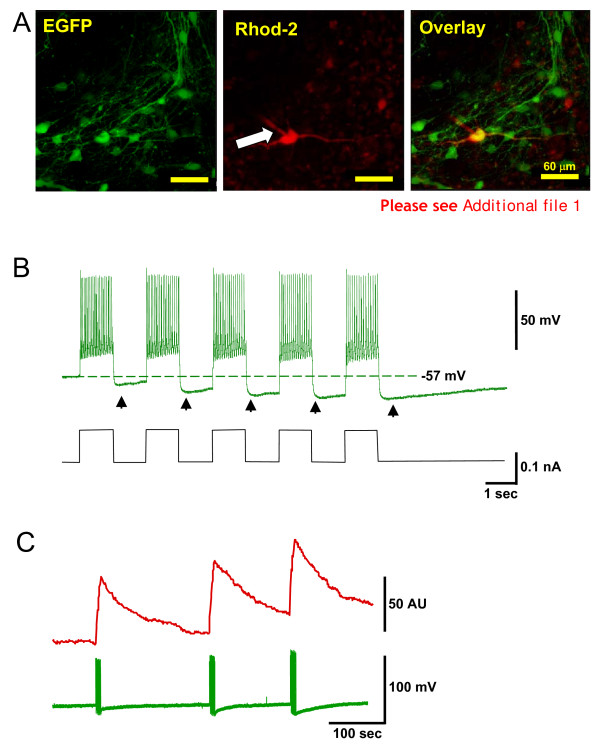
**Patch clamp and imaging of intracellular [Ca^2+^] in 5 HTergic neurones in slice culture**. **A**. Confocal image of EGFP-positive ventral raphe neurones (left panel), one of which was recorded in whole-cell configuration using a pipette solution containing Rhod-2 (centre panel; arrow indicates pipette tip). **B**. Representative recording from an EGFP-positive raphe neuron. Trains of action potentials (top trace) were triggered by injections of depolarizing current (lower trace). Note the prominent after-hyperpolarizations (arrowheads). **C**. Intracellular Ca^2+ ^dynamics in a 5 HTergic neuron based on Rhod-2 fluorescence (top trace; fluorescence intensity in arbitrary units, AU). Repeated trains of action potentials (bottom trace) induced by current injections resulted in reproducible [Ca^2+^] increases.

To confirm that the EGFP-expressing 5 HTergic cells were healthy, whole-cell patch clamp recordings were obtained from median raphe neurones using the same approach as in our previous studies [17,19] (Figure 3). The patch pipette contained the red-shifted Ca^2+ ^indicator Rhod-2 (Figure 3A, Additional file 1). Cells had membrane potentials in the range of -55 to -60 mV (n = 8, not corrected for the junction potential), and responded to positive current injections with barrages of action potentials. These were usually followed by long after-hyperpolarizations (Figure 3B–C), consistent with previous reports for raphe 5 HTergic neurones identified by post-hoc methods [32,33]. As expected, intracellular [Ca^2+^] increased when the cells were induced to fire bursts of action potentials (Figure 3C).

**Figure 4:**
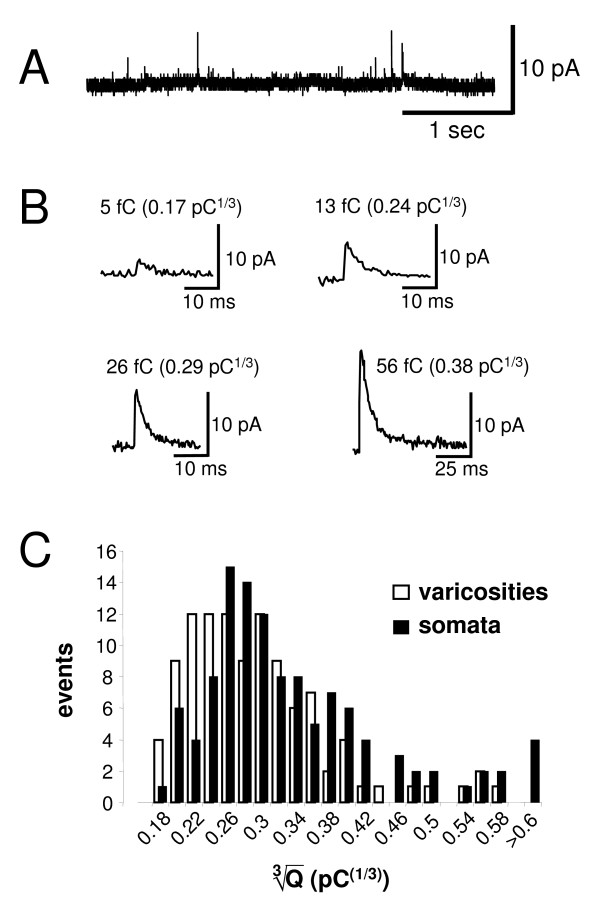
**Microamperometric analysis of 5 HT release in raphe slice culture**. **A**. Amperometric trace from an EGFP-expressing varicosity during an active episode. Spike-like events of varying sizes are visible. Note that the noise level looks exaggerated at this time scale due to low frequency baseline variability. **B**. Examples of amperometric spikes of various quantal sizes which were detected at 5 HTergic varicosities. **C**. Quantal size distribution of release events at 5 HTergic varicosities and cell bodies. At somata, several large release events, outside of the main population, were observed.

Vesicular release of oxidizable transmitters can be detected with micro-amperometry, an electrochemical technique which employs positively charged carbon fiber microelectrodes placed adjacent to release sites [31]. Oxidation spikes (Figure 4A–B), consistent with exocytotic release of 5 HT, were detected at fluorescently labeled varicosities and cell bodies of 5 HTergic neurones. The frequency of release events was low (average 0.01 Hz and 0.03 Hz for varicosities and somata, respectively). At varicosities, events had a median quantal size of ~18 fC, with a median amplitude of ~5 pA and duration at half-height of 3.1 msec (Figure 4C; 106 events from 5 release sites). At somatic release sites, the majority of events (97%) followed a similar distribution to varicosities, with a slightly higher median quantal content (~22 fC) and similar size and kinetics (~5 pA; 3.5 ms; 114 events in 5 recordings; Figure 4C). However, in addition, a few larger events were observed with a quantal content higher than 200 fC (average ~500 fC). Putative foot-like events, which are commonly interpreted as signs of partial fusion, were observed in less than 3% of events.

## Discussion

Most central 5 HTergic fibres originate from neurones concentrated within two clusters located close to the midline: a caudal group of the midline raphe nuclei, and a more rostral group that includes the dorsal and median raphe nuclei [34,35]. Raphe nuclei are often referred to as 5 HTergic but, in fact comprise a variety of neurochemical phenotypes. For example, both dorsal and medial raphe nuclei give rise to a glutamatergic projection towards the hippocampal formation [36] and glutamatergic neurones are intermingled with the TPH-2-positive cells which send their axons towards the same targets. Physiological roles of these parallel connections are very hard to disentangle without the help of new molecular tools such as the vectors described here. Interestingly, a generic "neuronal" promoter synapsin-1 is not suitable for targeting 5 HTergic neurones as the majority of the cells which expressed EGFP in animals injected with LVV-SYN-EGFP were non-TPH-positive [24]. Importantly, in slice cultures, vectors have access to any part of the brain tissue in contrast to *in vivo *micro-injections when their specificity (or lack of it) outside of the transduced area may remain unnoticed. The present results demonstrate that when AVV are applied *in vitro *they drive gene expression in 5 HTergic cells of various groups with very high specificity.

Because conventional AVV cannot incorporate very large transcriptional control units, we decided to place the TSTA-boosted 3.6 TPH promoter system into two separate rather than a single vector. Based on previous experience, we reasoned that the dual AVV system would function efficiently and greatly simplify vector design, leaving a generous capacity within the second vector. Here we only expressed EGFP, but in principle this system can easily accommodate larger transgenes such as bi-cistronic constructs or protein fusions.

EGFP fluorescence in present experiments was sufficient not only to visualize cell somata, but to reveal all the intricate detail of the 5 HTergic neurones' structure including axonal varicosities. From our earlier estimates [37], we believe that many EGFP-fluorescent 5 HTergic neurones expressed ~1 μM of EGFP. This for the first time made it possible to estimate the dimensions of living varicosities without the potential artefacts of fixation. Interestingly, we observed two distinct peaks in the size distribution of varicosities: the majority with a median size of approximately 1.5 × 1 μm and a minority category which is approximately 3 times larger. Whether these two populations of varicosities are functionally different is an intriguing question which needs to be addressed in future studies.

Functionally, EGFP-expressing 5 HTergic neurones had features consistent with what could be anticipated based on various previous studies where post-hoc immunohistochemistry or similar approaches were used. As shown in Fig. 3, these cells have healthy resting and action potentials, generate evident after-hyperpolarisations following a train of spikes and their intracellular Ca^2+ ^readily increases in response to stimulation. Thus, AVV do not have any evident detrimental effects on the transduced cells which survive in slice cultures for several weeks.

Although it was generally accepted that varicosities are the critical sites of 5 HT release, quantal characteristics of 5 HT release in the brain were previously unknown. By placing micro-amperometric electrodes on fluorescently identified cell compartments, we were able to collect initial information about this process. We found that 5 HT, alike central noradrenaline [31], is released by exocytosis not only from axonal varicosities but also from neuronal cell bodies. Because release from soma and dendrites occurs in the 5 HTergic nucleus itself while axonal release is directed towards the targets in remote brain areas it is tempting to speculate that the mechanisms which control release from these compartments could differ. Central 5 HT exocytosis occurred predominantly in quanta estimated at ~28000 molecules from varicosities and ~34000 molecules from cell bodies. In addition, in somata we observed a minority of large release events discharging on average ~800000 molecules. These categories of release events were comparable to the ones we previously recorded from central catecholaminergic neurones, as were their time courses [31]. However, the quantal sizes of 5 HT release were smaller than those of central catecholamine release. Also, frequencies of exocytotic events were markedly lower for 5 HT than for noradrenaline. Quantal release of 5 HT has been previously detected amperometrically from leech Retzius neurones [38,39] where two populations of release events, with average 5 HT discharge of 4700 and 80000 molecules, were postulated. Since these populations are consistent with release from the two different varieties of vesicles found in Retzius cells, namely small clear and large dense core vesicles (diameters ~40 nm and 90 nm based on ultrastructural data), 5 HT was suggested to be stored at intravesicular concentrations of around 0.27 M in both vesicle types [38,39].

Similar to Retzius cells, populations of small clear and large dense core vesicles (diameters ~40 nm and 100 nm, respectively) have been shown in central 5 HTergic and catecholaminergic neurones in the rat [40,41]. We found no convincing amperometric evidence for 5 HT release from a population of small clear vesicles. However, identification of some of the smallest release events could have been prevented by the noise limitations of our system. The main population of small release events in the rat raphe could represent release from large dense core vesicles, which should be either slightly smaller than in the leech (diameter ~70 nm versus ~100 nm), or contain a lower 5 HT concentration (0.1 M versus 0.27 M). Interestingly, the diameter of large dense core vesicles in leech somata was ~15% larger than in axons [39]. If the same applies to rat central 5 HTergic neurones, this could explain why our somatic events were ~7% larger than those recorded at the varicosities. For the very large 5 HT release events documented here, a morphological correlate remains to be found.

In terms of future directions, one evident path to explore is to express light-activated ion channels ChR2 and NpHR in 5 HTergic neurones [42,43] in order optically control their activity. Vectors may be constructed to express not only a fluorescent marker, but a functional gene, for example a 5 HT transporter. Although here we only describe *in vitro *applications of these novel AVV, they can also be used *in vivo*. An interesting property of AVV which we found in previous experiments with noradrenergic cells, is their ability to transduce cells via the retrograde route [9]. Because both, 5 HTergic and noradrenergic neurones have varicose un-myelinated axons, it may be possible that sub-groups of 5 HTergic neurones can be transduced by injecting AVV into their target areas. It would also be extremely interesting to see whether these AVV are suitable for targeting 5 HTergic neurones in other, non-rodent species.

## Conclusion

We have generated novel TSTA-amplified AVV suitable for selective gene expression in raphe 5 HTergic neurons of rodents and obtained the first key facts about quantal release of 5 HT in the brain. Numerous avenues are now open for further inquiries into the physiology of central 5 HTergic neurons and the mechanisms of action of drugs, such as blockers of 5 HT uptake, a widely used group of anti-depressants.

## Methods

### Construction of TPH-2 promoter-containing viral vectors

As described previously, lead promoter fragments were PCR amplified from the sequences 5' relative to the transcriptional initiation site of the rat brain TPH-2 gene, and inserted upstream of EGFP into a LVV shuttle plasmid [24]. These vectors also incorporated elements of TSTA. LVV were tested *in vivo *using microinjection into the rat raphe and from these experiments it was concluded that 3.6 TPH (promoter sequence starting 3.6 kb upstream of the coding region of the TPH-2 gene) drives highly specific and visible EGFP expression in 5 HTergic neurons *in vivo *[24]. In order to maximise space for expression cassettes, we placed the components of the TSTA system into two separate viral particles. To this end, the expression cassettes from the LVV shuttle plasmids pTYF-G4BS-3.6TPH-EGFP and pTYF-3.6TPH-GAL4|p65 were first transferred into the AVV shuttle plasmid, pXCX-Sw-linker, using *SceI *sites [44]. AVV were then produced by homologous recombination of shuttle and the helper plasmid pBHG10 in HEK293 cells. The media was collected for subsequent rounds of AVV proliferation in HEK293 cells until cytopathic effects were achieved. AVV were purified using CsCl gradient protocols [45]. Titers were established using an immunoreactivity spot assay as described previously [44].

### Viral vector transduction of dorsal raphe 5 HTergic neurons in organotypic slice cultures: Morphology, electrophysiology, electrochemistry

Organotypic coronal slice cultures of the rat pons/midbrain containing the raphe nuclei were prepared according to previously published protocols (thickness at the time of plating 250 μm) [15]. They were transduced with a mixture of AVV-G4BS-3.6TPH-EGFP and AVV-3.6TPH-GAL4|p65 at equivalent titers of ~2 × 10^8 TU/ml (2 × 10 μl of viral stock diluted into 1 ml of plating media) at the time of plating [15]. Cultures were used between 7 and 10 days *in vitro *when AVV-mediated EGFP expression was fully established. Imaging experiments were carried out at 33°C in a tissue chamber mounted on a Leica-SP2 confocal microscope. Amperometric recordings were carried out under similar conditions in a chamber based on a conventional inverted fluorescent microscope (Leica).

For morphometric studies Image Pro Plus (Media Cybernetics) software was used. Individual varicosities which could be clearly identified on confocal images taken from living tissue were processed to identify varicosities and obtain their dimensions.

Whole-cell patch clamp recordings in combination with confocal measurement of intracellular [Ca^2+^] changes were performed as described previously [17,19]. Rhod-2 was used as a Ca^2+ ^indicator to avoid spectral interference with EGFP.

Microamperometric recordings with carbon fibre electrodes manufactured in house were performed at a driving voltage of +800 mV, and analyzed as previously described [19,31]. Quantal sizes (Q in Coulomb) were derived from event integrals, and converted into the number of oxidized molecules by the equation [molecules = (Q × Avogadro's number)/n × Faraday constant), where n, the number of electrons transferred during oxidation of one transmitter molecule, is 4 for 5 HT [31,38,46].

### Immunohistochemistry

To assess the specificity of expression, slice cultures were immunostained for TPH-2. First, cultures were fixed on their membranes in 4% formaldehyde for 20 min, removed from the membrane and incubated in 10% horse serum with 0.3% triton in PBS for 1 hour. After washing 3× with PBS, the sections were treated with 50% ethanol for 30 minutes, and washed 3× for 30 minutes in 0.1 M PBS. They were then incubated for 72 hrs at 4°C in primary mouse monoclonal anti-TPH-2 antibody (Sigma, dilution 1:500), 5% horse serum and 0.3% triton in PBS. This step was followed by an overnight incubation with secondary donkey anti-mouse Alexa594 (Invitrogen, 1:300) and 2% horse serum. To ensure complete EGFP visualization, sections were counterstained with primary rabbit anti-EGFP antibody (Invitrogen, 1:2000), followed by an overnight incubation in the secondary donkey anti-Rabbit Biotin-SP-conjugated F(ab')_2 _IgG (Jackson ImmunoResearch, 1:500) and then in avidin-FITC for 4 hours. Sections were washed between incubations 3× for 30 min in PBS. Stained sections were mounted using Vectashield (Vector Labs), cover-slipped and imaged with a confocal microscope (Leica SP1) at 488 nm and 543 nm excitation for green and red channels, respectively. Channels were imaged sequentially using either 488 or 543 nm excitation to prevent "bleed" of the fluorescence between them.

## Abbreviations

5 HT: 5-hydroxytryptamine, serotonin; AVV: adenoviral vectors; CSP: cell-specific promoter; EGFP: enhanced fluorescent protein; LVV: lentiviral vector; PBS: phosphate-buffered saline; PCR: polymerase chain reaction; THP: tryptophan hydroxylase-2.

## Authors' contributions

KB generated the vectors, performed amperometric recordings and immunohistochemistry. BHL advised on cloning. AGT supervised cloning, micro-amperometry, analysed the data on quantal release and edited the manuscript. FT performed patch clamp and Ca^2+ ^studies. SK designed the study, analysed imaging data and wrote the manuscript.

## Supplementary Material

Additional file 1 **Patch clamp of a 5 HT neurone in organotypic slice culture.** Click here for file
